# Effects of a single bout of high-intensity-interval exercise on cardiovascular autonomic, cerebrovascular, and cognitive function in people with spinal cord injury: A study protocol

**DOI:** 10.1371/journal.pone.0326861

**Published:** 2025-07-01

**Authors:** Wenjie Ji, Jill M. Wecht, Hang Jin Jo, Filip Stefanovic, Jeffrey Miecznikowski, Nancy D. Chiaravalloti, Sue Ann Sisto

**Affiliations:** 1 Department of Rehabilitation Science, School of Public Health and Health Professions, University at Buffalo, Buffalo, New York, United States of America; 2 James J Peters VA Medical Center Department of Spinal Cord Injury Research, Bronx, New York, United States of America; 3 Icahn School of Medicine at Mount Sinai, Departments of Rehabilitation Medicine and Human Performance, New York, New York, United States of America; 4 Department of Biomedical Engineering, School of Engineering and Applied Sciences, University at Buffalo, Buffalo, New York, United States of America; 5 Department of Biostatistics, School of Public Health and Health Professions, University at Buffalo, Buffalo, New York, United States of America; 6 Kessler Foundation, Center for Traumatic Brain Injury Research, West Orange, New Jersey, United States of America; 7 Kessler Foundation, Neuropsychology & Neuroscience Research, West Orange, New Jersey, United States of America; 8 Department of Physical Medicine and Rehabilitation, Rutgers-NJ Medical School, Newark, New Jersey, United States of America; Huashan Hospital Fudan University, CHINA

## Abstract

**Background:**

Spinal cord injury (SCI) frequently disrupts the autonomic nervous system (ANS), impairing cardiovascular function and affecting cerebrovascular and cognitive functions. While high-intensity interval exercise (HIIE) is known to improve cardiovascular and cognitive functions in non-injured populations, its impact on these functions in individuals with SCI, especially those with high-level injuries, is not well-documented.

**Objective:**

The primary aim of this study is to investigate the acute effects of a single bout of HIIE on ANS related-cardiovascular (ANS-CV) function in individuals with chronic SCI at or above T6. The secondary aims are to examine the acute effects of the same HIIE bout on cerebrovascular dynamics and cognitive performance in this population.

**Methods:**

In this prospective case-control study, 15 individuals with SCI at T6 or above and 15 age- and sex-matched uninjured controls will be assessed. Measures include heart rate, heart rate variability, blood pressure, systolic blood pressure variability, cerebral blood flow velocities, and cognitive performance, analyzed pre- and post-HIIE. The sit-up test and face-cooling test will be used to activate the ANS-CV system. Post-exercise assessments will begin 5 minutes after completing the HIIE session. Cardiovascular testing will be conducted first and is expected to last 36 minutes. Cerebrovascular and cognitive testing will follow, starting approximately 41 minutes after the HIIE session. Covariates such as physical activity levels, pre-morbid intelligence, and psychological distress will be considered. This study has been approved by the University at Buffalo Institutional Review Board (IRB) (Approval Number: MOD00013354) and registered on ClinicalTrials.gov (Registration Number: NCT06274658).

**Results:**

We hypothesize that HIIE will improve cardiovascular and cerebrovascular functions and enhance cognitive performance in the SCI group. Data will be analyzed using linear mixed-effects models to evaluate the interaction effects of group and exercise.

**Conclusions:**

This study is expected to fill the knowledge gap regarding the impact of HIIE on cardiovascular, cerebrovascular and cognitive functions in individuals with SCI at or above T6. The findings will provide crucial insights into immediate physiological responses while establishing foundational evidence for developing targeted, long-term exercise interventions to improve health outcomes in this population.

## Introduction

Spinal cord injury (SCI) frequently disrupts the autonomic nervous system (ANS), leading to impairments in cardiovascular [1], cerebrovascular [2], and cognitive [3] functions. Individuals with a neurological level of injury (NLI) at or above the 6th thoracic vertebra (T6) are particularly vulnerable to more severe ANS-related cardiovascular (ANS-CV) disruptions [4]. While most anatomical studies localize cardiac sympathetic preganglionic neurons to the T1-T4/5 spinal segments [5,6], some evidence suggests occasional extensions to T6 [7,8]. This anatomical distribution aligns with observations that a SCI at or above T6 results in significant ANS-CV dysfunction [4], often manifesting as impaired blood pressure (BP) and heart rate (HR) regulation, including orthostatic hypotension (OH) and autonomic dysreflexia (AD) [9,10]. Physical inactivity following SCI may exacerbate ANS-CV dysfunction, underscoring the critical role of sustained activity in preserving ANS-CV function [11,12]. The downstream cognitive consequences of ANS-CV dysfunction are further complicated by interacting psychosocial factors. Cerebral blood flow (CBF) dysregulation may impair cognitive performance [13], but this relationship is nuanced by pre-morbid intelligence and psychological distress, which may independently influence cognitive outcomes [14–16]. Thus, a comprehensive assessment of ANS-CV, cerebrovascular and cognitive functions in SCI should account for physical activity level, psychological distress, and pre-morbid intelligence.

Given the potential interplay among ANS-CV, cerebrovascular, and cognitive dysfunction in individuals with SCI [13], interventions targeting improvements in ANS-CV function may also yield benefits for cerebrovascular and cognitive outcomes in this population. While it has been well established that exercise positively impacts ANS-CV, cerebrovascular and cognitive functions in non-injured populations [17–19], critical gaps persist in the SCI literature. Current exercise guidelines for SCI focus primarily on cardiometabolic and respiratory outcomes, with limited evidence regarding their effects on ANS-CV, cerebrovascular, and cognitive functions [20]. Specifically, only 8 studies have quantified exercise effects on direct ANS-CV parameters (e.g., baroreflex sensitivity [BRS], HR and BP variabilities) [12,21–27], and even fewer have examined cerebrovascular [28] or cognitive outcomes [28–30]. Importantly, these studies often employed inconsistent exercise intensity protocols and included heterogeneous participants (e.g., varying NLI) [12,21–30], making it particularly difficult to draw definitive conclusions about individuals with a NLI at or above T6. For example, a 6-month whole-body exercise program using hybrid functional electrical stimulation rowing led to improved BRS in individuals with a NLI at T1 to T10 [21]. However, only three out of the forty participants met the target HR [22], which raises uncertainty about the intensity applied or potentially reflecting individual variations in physiological capacity to increase HR. More recently, Ozturk and colleagues showed increased CBF velocity (CBFV) in SCI, which is a biomarker of cerebrovascular function, during cognitive tasks following long-term exercise training [28]. However, this study primarily addressed the duration of each exercise session rather than its intensity, making it challenging to develop detailed exercise guidelines.

Emerging evidence demonstrates that high-intensity-interval exercise (HIIE) offers superior cardiovascular, cerebrovascular and cognitive benefits compared to moderate-intensity continuous training in the general population [31–34]. Additionally, recent work in SCI specifically shows that moderate-to-high intensity arm cycling [12,26,27], but not low-intensity exercise [25], improves ANS-CV function, suggesting that adequate intensity is critical for physiological adaptation in this population. HIIE has emerged as a promising intervention for individuals with SCI, with prior studies demonstrating its feasibility and benefits for cardiorespiratory fitness in this population [35]. The time-efficient nature of HIIE directly addresses a key barrier to physical activity, while its intermittent structure may enhance adherence through greater enjoyment compared to continuous training [35]. While these studies have established the practical advantages of HIIE, its effects on ANS-CV, cerebrovascular, and cognitive functions remain unexplored. It is important to note that ANS dysfunction in people with a NLI at or above T6 might attenuate these benefits, as impaired sympathetic outflow blunts cardiovascular responses during exercise [36,37] and compromises cerebrovascular adaptation [38]. Further, impaired muscle and respiratory function in SCI may reduce physical fitness benefits such as maximal oxygen consumption [21], subsequently limiting hemodynamic and cerebrovascular adaptations to exercise [28]. Therefore, using exercise methods that accommodate preserved motor function in this population, along with inclusion of non-injured controls in studies, will allow direct comparison of HIIE effects in an intact versus ANS compromised cohort to help clarify how SCI influences adaptation to exercise.

We designed a rigorous prospective case-control study that includes a non-injured control group to bridge the knowledge gap regarding the impact of HIIE on ANS-CV, cerebrovascular, and cognitive functions in individuals with a NLI at or above T6. For this study, we selected arm cycling based on prior evidence of its acute benefits for ANS-CV function in SCI [26]. High-intensity exercise was defined as a rating of perceived exertion (RPE) ≥14 (Borg 6–20 scale) per ACSM guidelines [39]. Although ACSM also classifies intensity using HR (≥70% HR_max_), we prioritized RPE due to ANS dysfunction in SCI at or above T6, which may attenuate HR responses to exercises [36]. The primary aim is to evaluate the effects of a single bout of HIIE on ANS-CV function in individuals with a chronic SCI at or above T6. Secondary aims include assessing the impact of HIIE on cerebrovascular dynamics and cognitive performance in SCI. We hypothesize that an acute HIIE session will improve ANS-CV, cerebrovascular, and cognitive functions in SCI, but these benefits will be attenuated compared to non-injured controls due to ANS dysfunction. To account for individual variability, covariates such as physical activity level, pre-morbid intelligence, and psychological distress will be considered in the analysis.

## Methodology

### Study design

**Fig 1:**
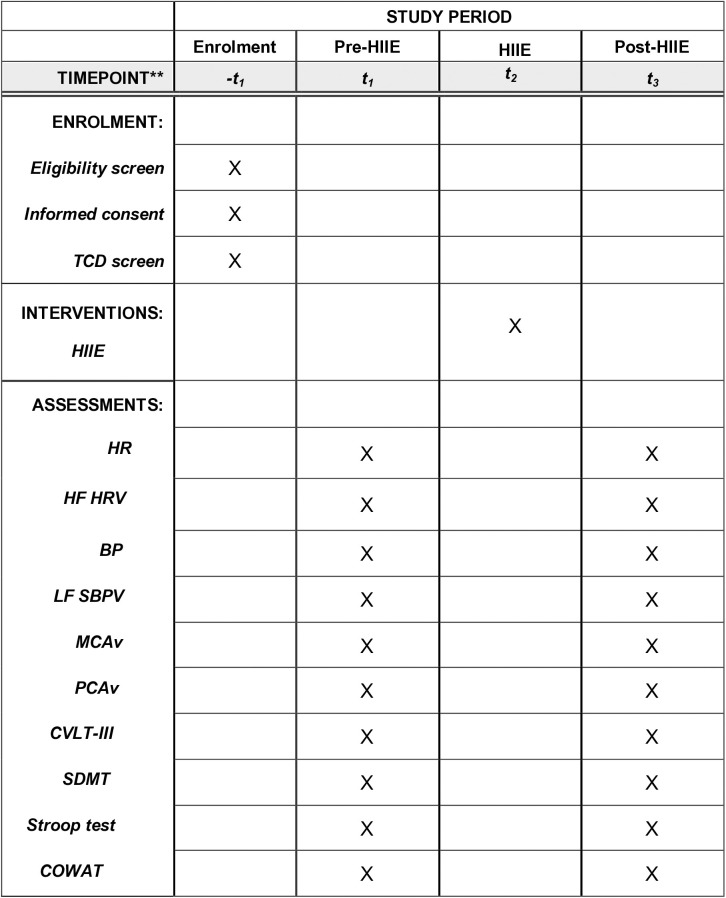
Schedule of Enrolment, Interventions, and Assessments. Notes: HIIE, high-intensity-interval exercise; TCD, transcranial Doppler; HR, heart rate; HF HRV, high frequency of heart rate variability; BP, blood pressure; LF SBPV, low frequency of systolic blood pressure variability; MCAv, middle cerebral artery blood flow velocity; PCAv, posterior cerebral artery blood flow velocity; CVLT-III, California Verbal Learning Test-Third Version, SDMT, Symbol Digit Modalities Test; COWAT, Controlled Oral Word Association Task.

This is a case-control study that includes a group of individuals with SCI and a sex- and age-matched uninjured control group to examine the effects of injury on exercise responses. The study protocol includes one initial visit for consent and maximal exercise testing (Visit 1) and one experimental visit (Visit 2) during which participants are exposed to a single session of HIIE. Fig 1 illustrates the schedule of enrollment, assessment, and intervention for the study (Fig 1).

This study has been approved by the University at Buffalo Institutional Review Board (IRB) on January 28, 2024 (Approval Number: MOD00013354) and registered on ClinicalTrials.gov (Registration Number: NCT06274658). The Principal Investigator (PI) will explain the study details to potential participants, and written informed consent will be obtained from all participants prior to their inclusion in the study.

### Sample size calculation

A pilot study was conducted to guide the design of this research. Based on the pilot data, we plan to recruit up to 20 participants per group, accounting for an estimated 20% attrition rate [40], to ensure at least 15 participants with complete data to be included in the analyses for sufficient statistical power (β ≥ 80%, α = 0.05). This sample size is calculated based on using a two-sample two-sided t-test to detect significant differences between the SCI and control groups for the change from baseline in ANS-CV function. High-frequency heart rate variability (HF HRV) reflects parasympathetic activity, with normalized units accounting for changes in total power during autonomic activation [41–43]. Similarly, low-frequency systolic blood pressure variability (LF SBPV; Mayer waves) serves as a biomarker of sympathetic vasomotor control [44]. Based on the pilot study, the estimated minimum detectable effect sizes are 24.97 for the difference in normalized HF HRV and 3.52 mmHg² for the difference in LF SBPV between groups, which are the two primary ANS-CV variables.

### Participants

Participants will be recruited from local rehabilitation clinics, fitness centers, and through word-of-mouth referrals. Participants will be eligible to participate if they meet the following criteria:

SCI Group

1. Diagnosed with traumatic or non-traumatic SCI with ≥4/5 strength in at least one C5 myotome (elbow flexors), enabling the use of an arm ergometer.
2. NLI at or above T6 [21].
3. Classified as A, B, C, or D (motor and sensory complete or incomplete) on the American Spinal Injury Association (ASIA) Impairment Scale (AIS).
4. At least 6 months post-injury and discharged to the community from inpatient rehabilitation prior to enrollment to ensure the stability of vital signs and minimize potential confounding factors associated with acute or subacute phases of SCI [45,46].
5. Aged 18–75 years as HIIE is considered relatively safe within this age range [47].
6. Proficient in English to ensure comprehension of the consent form and cognitive testing.
7. Detectable right middle cerebral artery (MCA) and/or left posterior cerebral artery (PCA) via transcranial Doppler (TCD) [48].

Non-Injured Group

1. Males or females without a history of SCI.
2. Aged 18–75 years.
3. Proficient in English to ensure comprehension of the consent form and cognitive testing.
4. Detectable right MCA and/or left PCA via TCD.

People showing interest will be screened for medical conditions that preclude exercise instructed by the ACSM’s Guidelines for Exercise Testing and Prescription, such as unstable angina, uncontrolled arrhythmias, recent untreated congestive heart failure, severe valvular disease, and uncontrolled hypertension [49]. Individuals with multiple sclerosis, diabetes, pre-existing shoulder injuries, or those who are pregnant will also be excluded to ensure safety during exercise and to obtain accurate HIIE outcomes on ANS-CV function in the proposed population. Given our focus on ANS-CV outcomes (HR/BP), we will ask participants to abstain from BP medications with direct hemodynamic effects on the day of testing. However, broader medication restrictions will not be mandated but medication names, doses, and purposes will be documented to assess potential medication-related influences on study outcomes.

### Experimental procedure

An initial screening will be conducted remotely through a demographic questionnaire sent via email or conducted over the phone. Interested individuals who meet all eligibility criteria except for detectable right MCA and/or left PCA will be invited for Visit 1. The laboratory ambient temperature will be maintained at 23°C, a thermoneutral range for humans [50]. This will minimize thermal stress risks in SCI participants, who are vulnerable to both hyperthermia and hypothermia due to impaired ANS regulation [4]. Before this visit, they will be instructed to abstain from medications with known effects on BP and HR for at least one-half-life of the medication(s) (with their physicians’ approval), as well as from caffeine, alcohol, and exercise for 24 hours [51]. Additionally, they will be asked to document their dietary intake and maintain their bowel routine. Upon arrival, participants will empty their bladder to limit reflex sympathetic activation. Then, informed consent will be obtained, followed by TCD screening. Two TCD probes will be placed on the temporal area using the transtemporal window to locate the right MCA and/or left PCA [40]. The probes will be secured with a headband, enabling continuous monitoring of CBFV during the trial. A photo of the setup will be taken to facilitate quicker identification of the MCA and PCA during Visit 2. Participants will be included in the study if the right MCA and/or left PCA are detectable. Following this, they will perform a graded exercise test to determine peak power output (PPO), calculated as the product of maximal resistance and the corresponding pedaling rate. Heart rate will be continuously monitored during the test using a Polar HR monitor for safety. Participants will continue to pedal within 25–60 revolutions per minute (rpm) until one of the following criteria are met: (i) volitional exhaustion or (ii) they are unable to maintain 20 rpm during 5 consecutive seconds or (iii) a rating of perceived exertion of 20/20 on the Borg scale [52]. Ratings of perceived exertion (RPE) will be monitored every minute. After the maximal exercise test, two questionnaires on psychological distress and physical activity level will be administrated and a pre-morbid intelligence test using the North American Adult Reading Test will be performed [53].

Visit 2 will be scheduled two to seven days after Visit 1 to allow for recovery from the maximal exercise testing and to ensure that there is no significant change in physical activity levels between the visits. Participants will receive the same pre-visit instructions for both visits, including maintaining a consistent diet, which is important due to its impact on ANS function [54]. Similar to Visit 1, participants will empty their bladder upon arrival to limit reflex sympathetic activation. They will then be seated, and the TCD probes will be secured to measure CBFV. Cognitive function will be evaluated with standard neuropsychological measures including the California Verbal Learning Test-III (CVLT-III) [55], Symbol Digit Modalities Test-oral version (SDMT) [56], Color-Word Interference Stroop Test [57], and the Controlled Oral Word Association Test (COWAT) to assess learning and memory, processing speed, executive function, and verbal fluency, respectively. These domains have been shown to be impacted in people with SCI [58,59]. Following cognitive testing, participants will undergo baseline cardiovascular measurements, including 15 minutes of beat-to-beat BP and electrocardiogram (ECG) recording, followed by a 1-minute face-cooling test (FCT) [60,61] to activate parasympathetic nervous system, a 5-minute recovery and a 15-minute sit-up test (SUT) [62] to activate sympathetic nervous system. To ensure participant safety during these tests and the subsequent HIIE protocol, continuous beat-to-beat BP and ECG monitoring will be conducted. If resting systolic BP exceeds 220 mmHg, the session will be immediately terminated based on a conservative safety threshold, which is well below the established 300 mmHg emergency criterion in SCI [63]. In cases of low BP, continuous monitoring will be maintained, and safety decisions will be guided by symptom presentation, as no universally accepted hypotensive threshold exists for this population.

**Fig 2:**
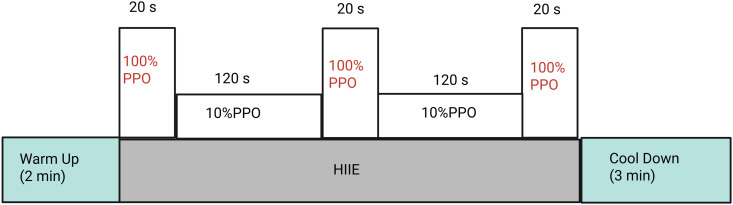
Overview of the High-Intensity Interval Exercise Protocol. Notes: PPO, peak power output.

Participants will then begin with a 2-minute warm-up at 10% PPO, followed by a single bout of HIIE. The HIIE consists of three 20-second all-out efforts at 100% PPO, each separated by 120 seconds of active recovery at 10% PPO. After completing the HIIE, participants will perform a 3-minute cool-down at 10% PPO [64]. This 10-minute protocol is one-third the time commitment of current exercise guidelines for SCI [20], and is therefore feasible for the targeted population. Fig 2 illustrates the HIIE protocol (Fig 2).

**Fig 3:**
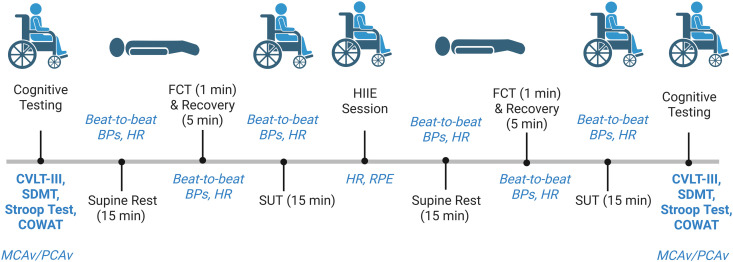
Study Procedures for the Experimental Visit. Notes: BP, blood pressure; COWAT, Controlled Oral Word Association Test; CVLT-III, California Verbal Learning Test Third Edition; FCT, face-cooling test; HIIE, high-intensity interval exercise; HR, heart rate; MCAv, middle cerebral artery blood flow velocity; PCAv, posterior cerebral artery blood flow velocity; RPE, rating of perceived exertion; SDMT, Symbol Digit Modalities Test; SUT, sit-up test.

After a 5-minute recovery (for photoplethysmography re-instrumentation), assessments will proceed within the peak ANS-CV modulation window (10–30 min post-exercise) [65]. The order prioritizes transient ANS-CV effects first, followed by cerebrovascular and cognitive assessments (after 41 min total), as exercise-induced cognitive benefits persist longer than cardiovascular effects [66]. Alternate forms of the CVLT-III, SDMT, and COWAT will be used for post-HIIE assessments to avoid familiarization effects. Fig 3 outlines the planned testing procedures for V2 (Fig 3).

### Measurements and tests

#### Physical activity and psychological distress questionnaire.

Physical activity will be assessed using the International Physical Activity Questionnaire-Short Form (IPAQ-SF) developed for the non-injured control group and the Leisure Time Physical Activity Questionnaire, which was developed specifically for People with SCI (LTPAQ-SCI) for the SCI group. The IPAQ-SF evaluates physical activity over the past 7 days, capturing vigorous, moderate, and walking intensities, as well as sedentary behavior, providing a comprehensive activity profile [67]. The LTPAQ-SCI is an SCI-specific tool measuring mild, moderate, and heavy intensity leisure-time physical activity over the same period, with a structure similar to the IPAQ-SF [68,69]. Both self-administered questionnaires are validated, widely used, and take less than 5 minutes to complete [67–69]. Psychological distress will be evaluated using Hospital Anxiety and Depression Scale (HADS) designed to identify symptoms of anxiety and depression in both the general population and individuals with SCI [70–72]. The HADS consists of 14 items, evenly divided into two subscales: anxiety (HADS-A) and depression (HADS-D). Each subscale contains 7 items, with participants rating their experiences over the past week on a 4-point Likert scale. This tool provides a reliable and efficient evaluation of psychological distress, making it suitable for use in clinical and research settings [70–72].

#### Cardiovascular function.

**Sit-up test (SUT)***:* The SUT will be conducted using a wheelchair equipped with a reclining function down to 0 degrees (supine). Participants will initially be positioned in the reclined wheelchair for 15 minutes. Subsequently, they will be gently assisted into a seated position, with their feet placed on the floor, and asked to maintain this posture for another 15 minutes. During this seated phase, participants will be monitored for BP and HR, and questioned for symptoms of orthostatic hypotension, such as blurry vision, dizziness, light-headedness, or nausea. HRV and SBPV will also be obtained afterwards through the original BP and HR data. The SUT is recognized as a reliable method to induce an orthostatic challenge in individuals with SCI [62].

**Face-cooling test (FCT)**: A pliable plastic bag filled with 2.5 L of ice water (~0°C) will be placed on participants’ forehead, the bridge of the nose, and cheeks for 1 min. A duration of one-minute FCT activates the trigeminal nerve, leading to a transient surge in cardiovascular parasympathetic activity and bradycardia [73]. During the FCT, BP, HR, HRV, and SBPV will be assessed to monitor these cardiovascular responses.

#### Cerebrovascular function.

Cerebrovascular function will be assessed using CBFV during cognitive testing (see the Cognitive Function section below). In order to ensure that hemispheric differences in velocity does not inﬂuence the results, which do occur in the MCA and PCA [74], CBFV will be consistently recorded in the right MCA and the left PCA (P1 segment) during cognitive testing.

#### Cognitive function.

**North American Adult Reading Test (NAART)**: The NAART consists of a list of 61 words printed in order of increasing difficulty. The words are relatively short to avoid the possible adverse effects of reading stimulus complexity, and they are all ‘irregular’ with respect to the common rules of pronunciation to minimize the possibility of reading by phonemic decoding rather than word recognition. The subject would read aloud down the list of words and the number of errors made is recorded. Premorbid intelligence can be predicted by the NAART score [53].

**Stroop test**: The Stroop Test consists of three sections including: (1) Word Reading (WR), where subjects will be required to read three words (red, green, blue), printed in black ink, in a randomized and repeated manner as quickly as possible; (2) Color Naming (CN), involving the naming of the color of a group of Xs (XXXX) presented in red, green, and blue ink; and (3) Inhibition, on which subjects will be asked to identify the ink color of printed color names while disregarding the word itself (i.e., the word red printed in blue ink should be identified as blue). Both the WR and CN assess processing speed, while the inhibition task evaluates cognitive inhibition. Each task will be performed for a duration of 45 seconds, with subjects responding verbally as fast as possible. Any incorrect responses will result in interruptions, adding to the erroneous time and thus reducing the score. The raw score, denoting the number of correct responses within 45 seconds, will be compared against predicted values based on the individual’s age and educational level. The deviation between the actual and predicted scores will be calculated and then converted into age and education normative t-scores [38].

**Symbol Digit Modalities Test -Oral Version (SDMT)**: The SDMT-oral version is included as a supplementary measure of processing speed that does not have a motor component. It has been demonstrated to be sensitive to the presence of brain damage in numerous studies. The SDMT requires the subject to substitute a number for a randomized presentation of a geometric figure. The appropriate number is shown in a key containing the Arabic numbers 1–9, each with a different geometric figure [75]. As the examinee looks at each stimulus, they state the number that is paired with each symbol in the key. The SDMT will be administered over 90 seconds, with the number of correct responses recorded.

**California Verbal Learning Test-III (CVLT-III)**: The CVLT-III assesses verbal learning and memory. It consists of 16 words from 4 semantic categories presented orally over 5 trials and includes a 20-minute delayed recall trial and a recognition trial [76]. The normed score for each trial will be calculated based on participants’ ages and educational levels.

**Controlled Oral Word Association Task (COWAT)**: The COWAT is designed to provide a score representing verbal fluency. Participants are asked to generate and pronounce as many English words as possible beginning with each of the letters C, F, L (or F, A, S) for an alternate version post-exercise) in three serial trials or tasks lasting 1 minute each [77]. The score is calculated by summing the total number of correct words generated for each letter, across all three trials [78]. The total score represents the participant’s verbal fluency, with higher scores indicating better performance.

### Equipment

**Data acquisition:** BIOPAC Systems, Inc. MP160 system will be used for data acquisition and analysis of physiological data. The MP160 is a 16-channel core system with a high-level transducer interface module that uses AcqKnowledge 5.0 software for visualization and processing. The specifications for each data modality are described below.

**Baroreflex sensitivity analysis:** BRS will be processed using transfer function analysis via MATLAB [79]. BRS is represented by the average gain between changes in systolic blood pressure and the R-R interval within the frequency range of 0.07–0.14 Hz, a range that is reliable for BRS assessment as it is not significantly influenced by respiratory rate [79]. The beat-to-beat BP data will be down-sampled to 250 Hz from 1000 Hz to align with the ECG dataset for this analysis [80].

**Cerebral blood flow velocity**: The Lucid M1 TCD system (NovaSignal, Los Angeles, CA), will be used to measure CBFV. The ultrasound probe will be secured over the temporal acoustic window using an adjustable headpiece to ensure stable and consistent signal acquisition. The sampling rate will be set at 1000 Hz.

**Electrocardiogram:** Bionomadix 3-lead ECG (DA100C, Biopac Systems, Goleta, CA) will be used to obtain the R-R interval. Electrodes will be attached after preparing the skin with an alcohol wipe to reduce impedance and improve signal quality. The sampling rate will be set at 250 Hz.

**Heart rate variability:** LabChart 8 Software with built-in heart rate variability (HRV) tools will be used for ECG clean-up, including a QRS detector, beat-to-beat analysis, and autocorrection for ectopic beats. High-frequency (HF; 0.15–0.45 Hz) HRV will be calculated from the spectral analysis of the R-R interval through LabChart 8 software [81]. Normalized HF HRV (HF HRV n.u.) will be calculated by dividing raw HF by (raw HF + raw LF HRV).

**Photoplethysmography:** ClearSight photo-plethysmograph (Edwards Lifescience Inc, Irvine, CA; formerly Nexfin BMEYE, Amsterdam, Netherlands), will be used on the index or middle finger to obtain blood pressure waveforms. As a quality control measure, calibration using the Physiocal™ vascular unloading algorithm will be used prior to each physiological test. The sampling rate will be set at 1000 Hz.

**Systolic blood pressure variability:** SBPV will be processed using the methods previously reported in MATLAB [44]. Occasional ectopic beats will be removed by substituting them with average values of the resting condition, and significant trends will be eliminated by subtracting the best-fit polynomial function. The low frequency (LF; 0.05–0.15 Hz) band will be identified using Fast Fourier Transform [44]. Subsequently, the absolute power of the LF SBPV will be calculated using power spectral density.

### Outcome measures

**Primary outcome measures**: The outcome measures for our primary objective will include HF HRV, LF SBPV, BRS, HR, and BP during supine assessments, FCT, and SUT, both pre- and post-a single bout of HIIE.

**Secondary outcome measures**: The outcome measures for our secondary objective will include CBFV during cognitive testing and cognitive performance assessed pre- and post-a single bout of HIIE.

### Data analysis

To analyze the impact of HIIE on cardiovascular, cerebrovascular, and cognitive functions in individuals with SCI compared to controls, linear mixed-effects models (LMMs) will be utilized. These models will assess the main effects and interactions of group (SCI vs. controls) and time (pre-HIIE vs. post-HIIE) across different outcomes, with specific adjustments tailored to the type of function being analyzed. The general model can be structured as follows:

Y_ij_ = β_0 _+ b_i_ + β_1_Group_i_ + β_2_Time_j _+ β_3_Group_i _× Time_j _+ β4 × Covariates_i _+ ∊_ij_

Where:

- Y_ij_ represents the dependent variable (e.g., HR, BP, HF HRV, LF SBPV, BRS) for subject *i* at time j (j = 0,1).
- Group_i_ is the SCI/control status for subject *i* (SCI: Group_i _= 1, Control: Group_i_ = 0).
- Time_j_ is the *jth* time point (pre-HIIE: Time_j_ = 0, post-HIIE: Time_j_ = 1).
- Covariates_i_ are additional covariate(s) for subject *i*. For dependent variables within cardiovascular and cerebrovascular functions: baseline physical activity levels. For cognitive function: pre-morbid intelligence and psychological distress.
- b_i_ is the random effect for subject *i* to account for within-subject correlation over time and is assumed independent N(0, σ_b_^2^).
- ∊_ij_ is the residual error term assumed independent N(0, σ_∊_^2^).

Estimation of parameters will be analyzed by restricted maximum likelihood estimation (REML). Wald tests will be used to assess the significance of the predictors, with the significance level set at p < 0.05. All analyses will be conducted using RStudio (© 2009–2024 Posit Software, PBC).

### Data management

The investigators will implement measures to ensure participant anonymity by utilizing deidentified data. The identification key linking participants to their study identifiers will be securely maintained and accessible only to authorized study personnel. The data will be overseen by the University IRB. All signed informed consent forms will be retained for the IRB-mandated period of three years following the conclusion of the study. Any adverse events reported during the intervention will be promptly communicated to the University IRB.

### Study status

The initial IRB approval (Version 1) date was January 28, 2024. Study recruitment began on February 14, 2024, with an anticipated completion date of May 30, 2025. Data collection is currently ongoing.

### Data

Deidentified data will be made available upon reasonable request to the Principal Investigator (PI) and solely for scientific review purposes. Access to the data will be granted only after the results have been published in peer-reviewed journals.

## Discussion

High-intensity interval exercise (HIIE) as a therapeutic intervention for individuals with SCI has emerged as a promising area of research [35], particularly in its potential to address disruptions in ANS function [12,26]. While broader benefits of exercise for cardiovascular and cerebrovascular health are well-documented in the non-injured population [82,83], its specific effects on individuals with SCI, particularly those with a NLI at or above T6, remains poorly understood. Current evidence lacks normative values for ANS-CV and cerebrovascular parameters in SCI. While LF SBPV less than 2 mmHg^2^ predicts OH [44] and higher diastolic CBFV correlates with better cognitive performance [84], absolute thresholds defining ANS-CV or cerebrovascular dysfunction remain undefined for this population. While this acute cross-sectional study cannot determine definitive efficacy endpoints or minimum clinically important differences (MCIDs) for HIIE in SCI, the findings will identify acute physiological responses that may predict clinically meaningful outcomes. In particular, we will evaluate pre- to post-HIIE changes in the ANS-CV, cerebrovascular, and cognitive variables between the SCI and control groups. If the direction and pattern of post-HIIE responses in the SCI group resemble those observed in the control group, this may be indicative of adaptive or beneficial changes, even if absolute response magnitudes are blunted. Additionally, the outcome measures obtained from the control group may provide preliminary normative data to inform future investigations in this field. This is particularly relevant given the unique challenges faced for people with SCI, including impaired BP and HR regulation, and decreased cognitive performance, all of which can profoundly impact quality of life [13].

Previous studies have highlighted the benefits of long-term exercise regimens on ANS and cognitive function in individuals with SCI, possibly through ventricular remodeling, enhanced myocardial contractility, improved BRS, improved respiratory function, and increased CBF [12,21,24,28,30,82,85]. Additionally, the potential benefits of exercise on ANS-CV function in individuals with SCI may be mediated by non-neuronal mechanisms, such as increased circulating catecholamines that enhance cardiovascular responses to exercise, despite impaired sympathetic innervation to the heart [86]. An exaggerated chemoreflex sensitivity, commonly observed in SCI, may also contribute to these effects [87], along with residual pressor responses to exercise, particularly in those with higher-level injuries [88]. Together, these compensatory pathways may help explain observed improvements in ANS-CV function following exercise, even in the presence of disrupted descending sympathetic control. The current investigations on ANS-CV, cerebrovascular, and cognitive functions in SCI primarily focused on moderate-intensity or long-duration interventions, leaving a critical gap in understanding the impact of intensity-focused, time-efficient protocols such as HIIE, especially since lack of time is a significant barrier to physical activity among individuals with SCI [89]. Acute HIIE has been shown to improve wall shear stress and endothelial function in obese or sedentary individuals [90,91], which may subsequently benefit ANS-CV, cerebrovascular, and cognitive functions, though these mechanisms require further investigation in people with SCI [92–94]. Moreover, existing research has often generalized findings across participants with varying NLIs, limiting the applicability of results to individuals with more severe cardiovascular dysfunction associated with higher-level injuries [12,21,24,28,30].

This study protocol addresses these gaps by focusing on the acute effects of HIIE, a short-duration yet high-impact exercise modality, on ANS-CV, cerebrovascular, and cognitive outcomes in individuals with SCI at or above T6. Unlike previous exercise studies in SCI [12,21,22], this protocol incorporates sensitive ANS-CV tests, such as the SUT and FCT, rather than relying solely on resting conditions. This approach provides a more dynamic and comprehensive understanding of how HIIE influences physiological and cognitive functions in this population. Furthermore, this study incorporates critical covariates (i.e., physical activity levels, pre-morbid intelligence, and psychological distress) to ensure robust and nuanced analyses. By addressing these variables, the study aims to generate findings that are both clinically relevant and generalizable. This approach is particularly innovative, as previous studies have largely overlooked the interplay between these covariates and exercise-induced physiological changes in the SCI population.

The implications of this research extend beyond the immediate outcomes. By demonstrating the feasibility and potential benefits of HIIE, this protocol sets the stage for larger, long-term studies that aim to refine exercise prescriptions for individuals with SCI. Additionally, the use of advanced physiological monitoring techniques could pave the way for more personalized exercise interventions tailored to the unique needs of each individual, based on their ANS-CV, cerebrovascular, and cognitive profiles.

### Limitations

There are several inherent limitations in the study protocol. First, the research will utilize TCD to quantify CBFV. Notably, challenges in TCD data acquisition have been documented among certain demographics, including blacks, Asians, and elderly women, primarily due to a 10–15% prevalence of an inadequate acoustic window [40]. In our previous study (not published), despite performing TCD screenings at the consent stage to ensure the detectability of cerebral vessels, the MCAs were still not detectable in two of the six participants during subsequent experimental sessions. To mitigate this, photographic documentation of each participant’s MCA/PCA location will be captured during initial screenings. This strategy aims to expedite and ensure accurate MCA/PCA localization in subsequent experimental sessions. Second, participants will provide self-reported information regarding their NLI. It is important to acknowledge that this approach may introduce the possibility of inaccuracies arising from potential memory lapses and changes in NLI due to natural recovery affecting motor and sensory functions. To mitigate this limitation, the study will exclusively enroll individuals with chronic injuries, defined as those with an onset of injury exceeding 6 months. Previous evidence has indicated that NLI tends to stabilize after this period following the initial injury [95]. Third, it’s pertinent to highlight that the scope of this study is restricted to particular parameters, excluding potential influences such as partial pressure of carbon dioxide level [96], sleep-disordered breathing [97], concurrent mild traumatic brain injury [3], or specific dietary impacts [98]. Subsequent research studies should consider these aspects to provide holistic insight into cognitive function post-SCI and the impact of exercise training. Fourth, due to the inconsistencies in current evidence regarding menstrual cycle effects on autonomic and cardiovascular responses [99,100], our study does not control for cycle phase. We recommend future research record basic menstrual cycle information (e.g., self-reported phase) to help contextualize individual response variability in training adaptations in females with SCI. Fifth, thermoregulatory compromise was not addressed in this study due to the brief duration of the HIIE protocol (10 minutes) and the controlled laboratory setting. However, we acknowledge that thermoregulatory challenges, particularly in individuals with NLI at or above T6, may compromise exercise responses in real-world applications. Future studies should standardize ambient temperature (20°C–25°C) and relative humidity (30%–50%) to mitigate such risks. Sixth, this protocol does not control for respiratory rate during the FCT, as common respiratory rate primarily affects HF HRV. However, previous evidence suggests that FCT induces minimal changes in respiratory rate [101]. Additionally, requiring participants to maintain a controlled respiratory rate could introduce unnecessary cognitive distraction, potentially affecting ANS function [102]. We therefore do not consider this a significant limitation of the study. Finally, this study does not assess post-exercise subjective fatigue. Although fatigue may influence cognitive performance during post-HIIE assessments, a validated fatigue scale (e.g., visual analog scale) was not included in the current protocol due to the timing of study initiation. Future studies should consider incorporating brief fatigue assessments to better differentiate cognitive effects that are exercise-induced versus those related to acute exertion.

### Dissemination plans

The results of this trial will be presented at national and international conferences and will be published in peer-reviewed journals.

### Study amendments and termination

Any amendments to the study protocol will be reviewed and approved by the University IRB before implementation. Participants will be informed of any changes that may affect their involvement in the study. In the event of study termination, a detailed rationale will be documented, and the IRB will be notified immediately. The IRB is responsible for protecting the rights and welfare of trial participants, evaluating the safety and efficacy of interventions throughout the trial, and monitoring the trial’s overall conduct. Data collected up to the point of termination will be securely stored and analyzed according to the approved protocol.
